# Supplementary material for: Integrated Metabolome and Transcriptome Analysis Provide Insights into the Effects of Grafting on Fruit Flavor of Cucumber with Different Rootstocks
Source: Int J Mol Sci. 2019 Jul 23;20(14):3592. doi: 10.3390/ijms20143592 (PMC6678626; doi:10.3390/ijms20143592)
Supplement: Supplementary file 1 [file ijms-20-03592-s001.zip › supplement data/Table S5.docx]

| Term | KEGG ID | Input number | Background number | P-Value | Corrected P-Value | Treatments |
| --- | --- | --- | --- | --- | --- | --- |
| Diterpenoid biosynthesis | ko00904 | 3 | 38 | 0.00075745 | 0.01590643 | GNo.45-NG |
| Carotenoid biosynthesis | ko00906 | 2 | 31 | 0.00968764 | 0.20344042 | GNo.45-NG |
| Photosynthesis - antenna proteins | ko00196 | 2 | 19 | 0.00369031 | 0.07749648 | GNo.45-NG |
| Plant hormone signal transduction | ko04075 | 6 | 271 | 0.00107873 | 0.02049595 | GNo.96-NG |
| Diterpenoid biosynthesis | ko00904 | 4 | 38 | 0.00049875 | 0.01645875 | GNo.45-GNo.96 |
| Plant hormone signal transduction | ko04075 | 8 | 271 | 0.00468486 | 0.15460052 | GNo.45-GNo.96 |

Table S4 Significant pathways in cucumber fruit as identified by KEGG analysis in different comparison combinations.
