# Supplementary material for: Integrated Metabolome and Transcriptome Analysis Provide Insights into the Effects of Grafting on Fruit Flavor of Cucumber with Different Rootstocks
Source: Int J Mol Sci. 2019 Jul 23;20(14):3592. doi: 10.3390/ijms20143592 (PMC6678626; doi:10.3390/ijms20143592)
Supplement: Supplementary file 1 [file ijms-20-03592-s001.zip › supplement data/Table S1.docx]

**Table S1. Genes primer used for RT-qPCR in self-graft cucumber, cucumber grafted onto different rootstock by transcriptome analysis.**

| **Gene name** | **Forward primer （5’- 3’）** | **Reverse primer （5’- 3’）** |
| --- | --- | --- |
| Csa7G071610 | TGGGAGGATTGCGAGGAGA | AAGTGATATGCCATTGTCAGC |
| Csa3G402970 | CATCGGCATACCAGTTCG | GGCTTTCCTGTCCCATTT |
| Csa5G568300 | AGAGGAGGCGAGGAAGGGAA | GTGGCTGATTGGAAAGTTGTTGA |
| Csa7G343850 | CCCTTCGTGTTGGCTCA | TACGGCTGCTGCGTTTC |
| Csa4G028470 | GAGCCAAAGTGGAGAACA | TGGGTTAGTCGGAGCAT |
| Csa1G611290 | CAACATCTTGGCTTGCTAT | TATCCCTTTACCTTTACGC |
| Csa6G500540 | GCTAAGGTGGAGAACAACA | TCTTCCTTGGGTTAGTCG |
| Csa2G252020 | AATCCCTCCTGCTGTTCC | CATCCTGTGCGATCTTCAC |
| Csa2G023880 | GTTAGCAGGACCCAATCC | CCTCATCAACCGTAAGACC |
| Csa6G487590 | TGGGATTAGCATCTGGC | CATTGGCGTTGGCATA |
| Csa2G404730 | ACGCCGCTTACATCTCC | AGGCAAGCAAGACCACC |
| Csa2G004690 | TCTGGCGGTGGATTGA | ATGGATAAGGCGGAGGC |
| Csa3G119240 | CTAGGCTTGAGCCCAGAA | TGGACCGCTGTACCAGAT |
| Csa3G904140 | AGGTTTGACAAGGCACAA | GGATGATAAGAAGGGTAAGC |
| Csa5G434550 | AGCAAACGGACTCCAAA | CAGCTCCTTCTTCCAAGC |
| Csa2G004690 | TCTGGCGGTGGATTGA | ATGGATAAGGCGGAGGC |
| Csa4G630010 | AATACGGCAGAAGAAGCG | GCAAGCGGATTTATGGA |
| Csa3G822100 | CATCGCTAAGAAACATACCA | CAGTTTATTCACATCGCACA |
| Csa1G703060 | CTACGGAAGCGGAAGCA | CGTCTGCGAGGGCTAAA |
| Csa1G589710 | TTCCGACGAGCATTCC | GTAGCCAACCGCCATAT |
| Csa1G015720 | GGGTGTACCAGTTACTCCAA | GCCTCTTTCTCGCCATT |
