# Supplementary figures and images for: Integrated Metabolome and Transcriptome Analysis Provide Insights into the Effects of Grafting on Fruit Flavor of Cucumber with Different Rootstocks
Source: Int J Mol Sci. 2019 Jul 23;20(14):3592. doi: 10.3390/ijms20143592 (PMC6678626; doi:10.3390/ijms20143592)

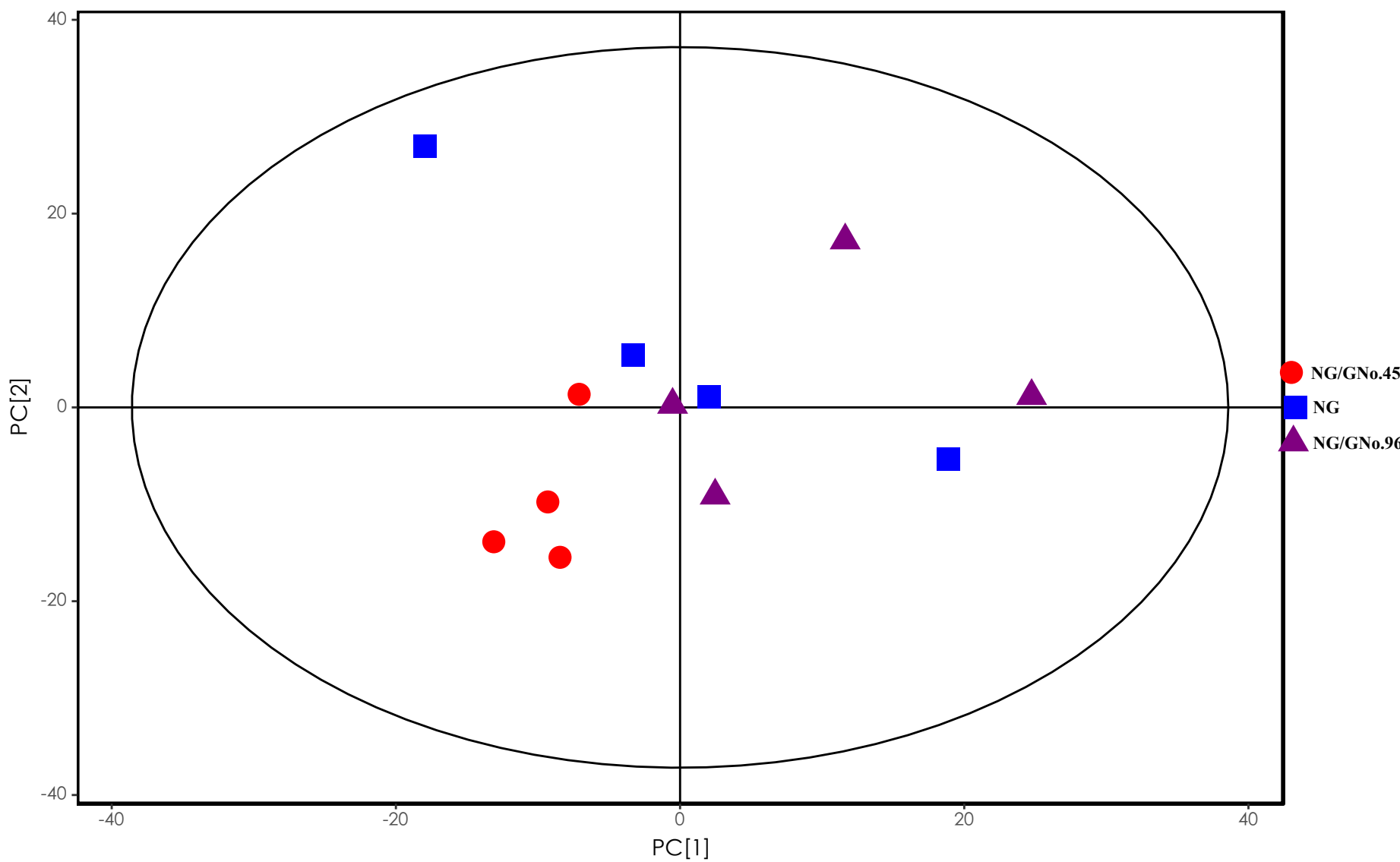

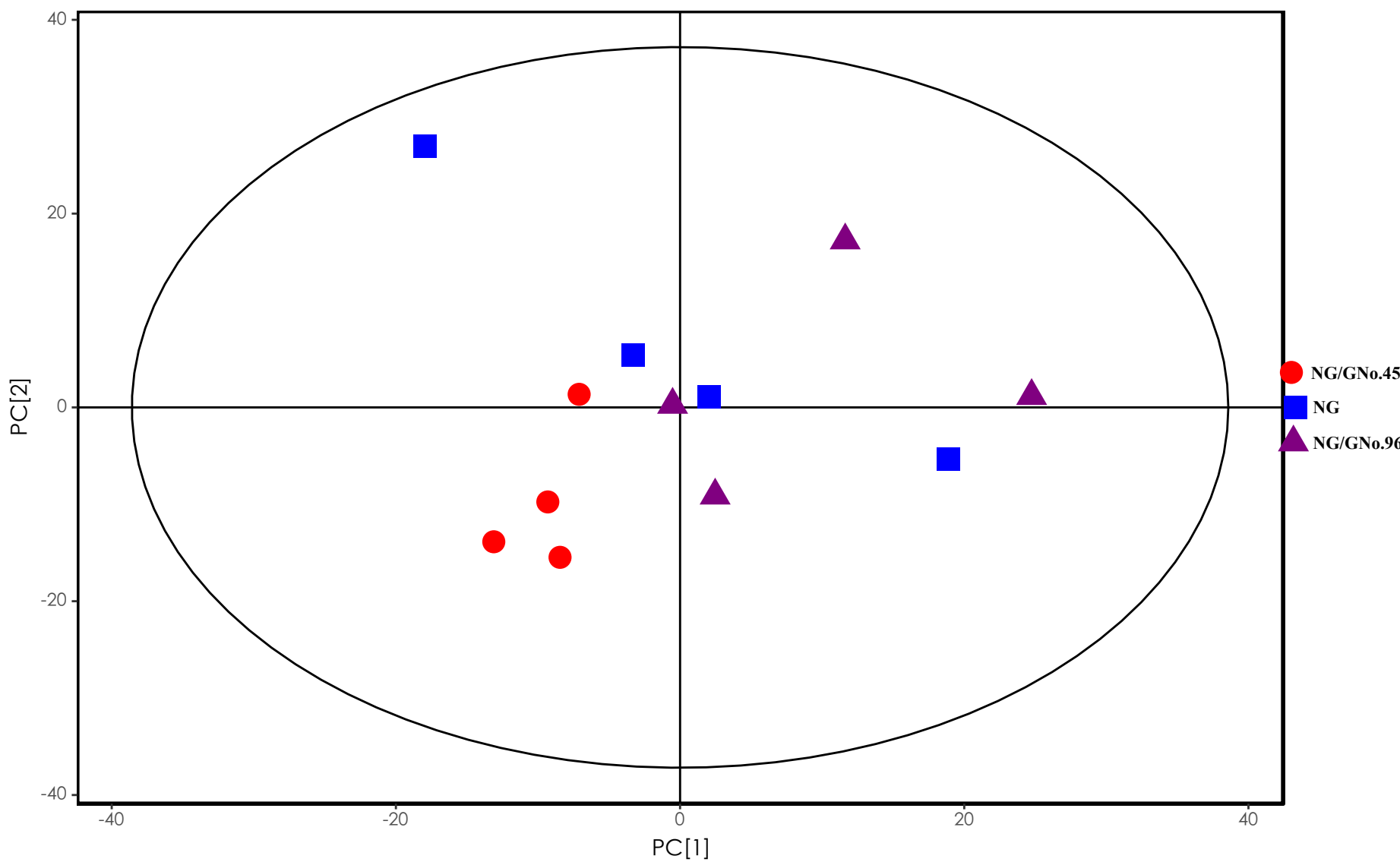

Supplement: Supplementary file 1 [file ijms-20-03592-s001.zip › supplement data/Figure S1.pdf]

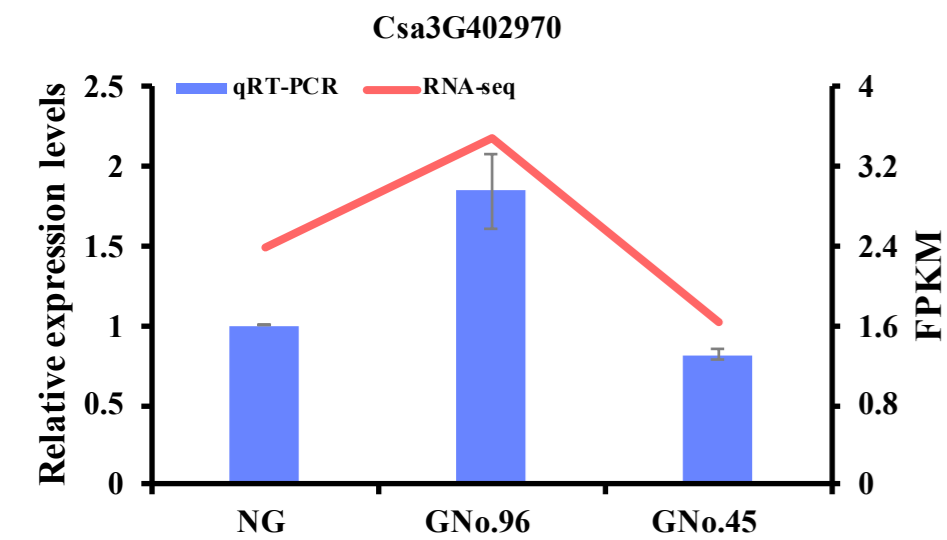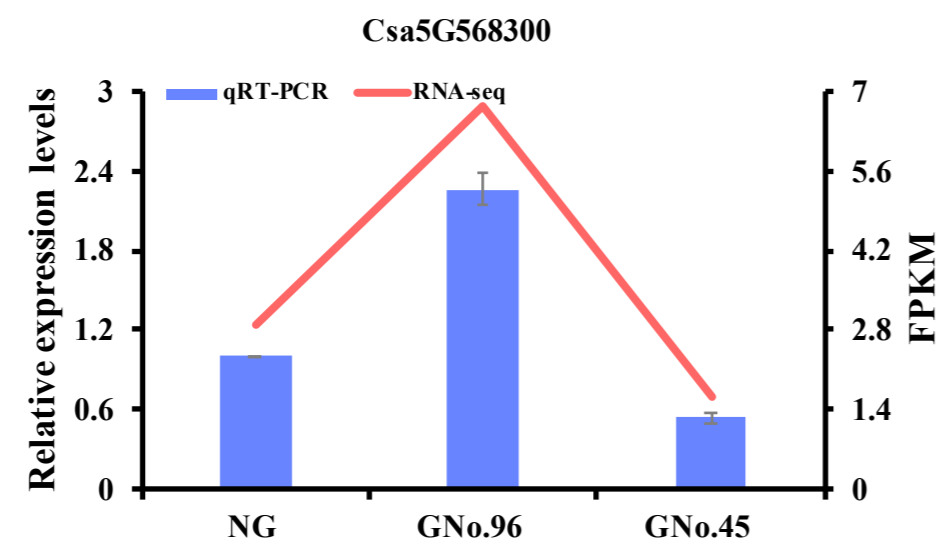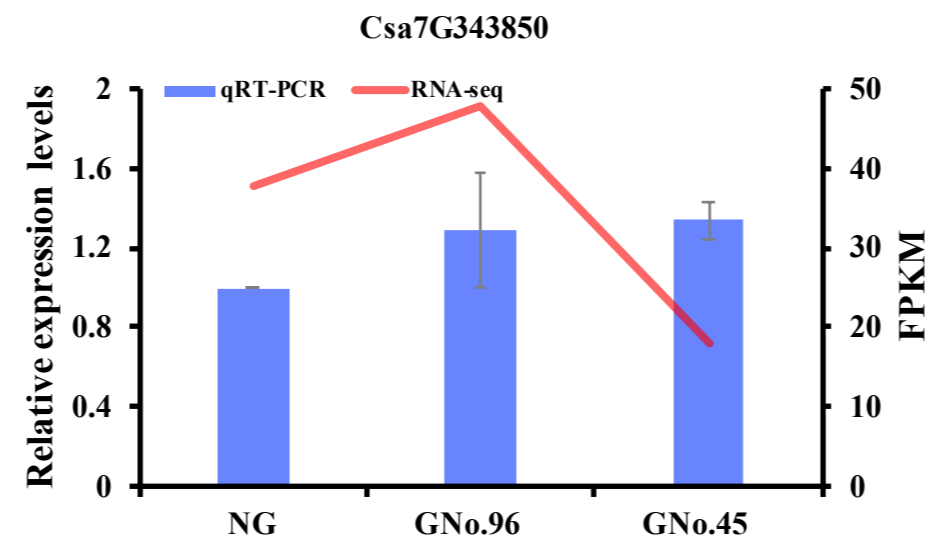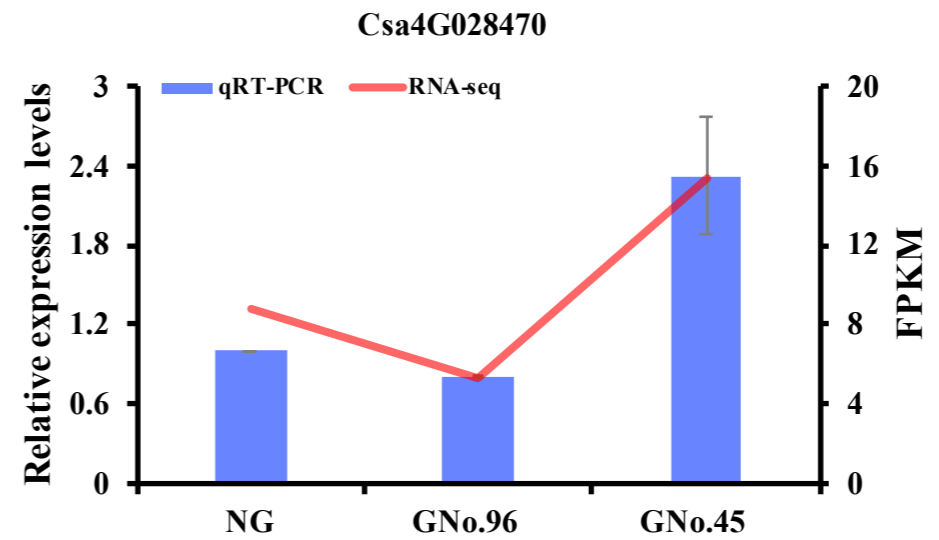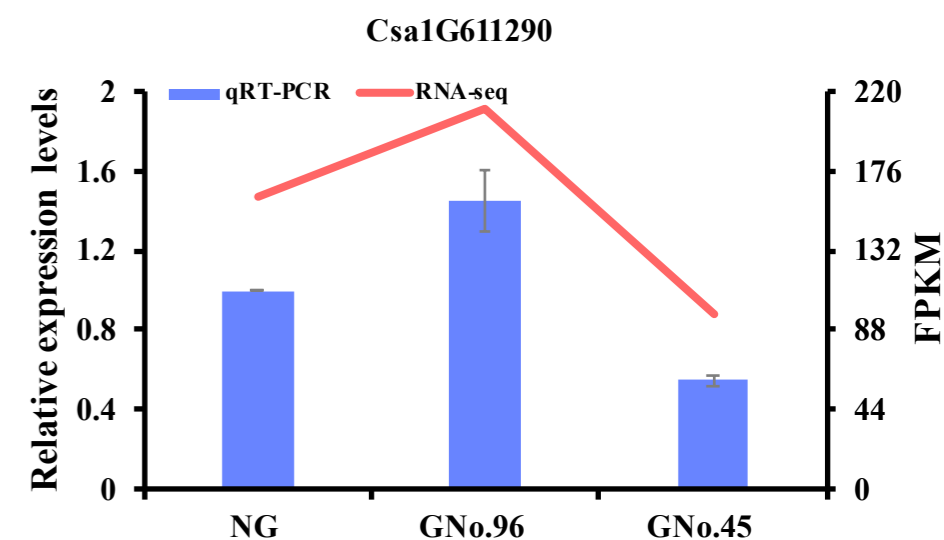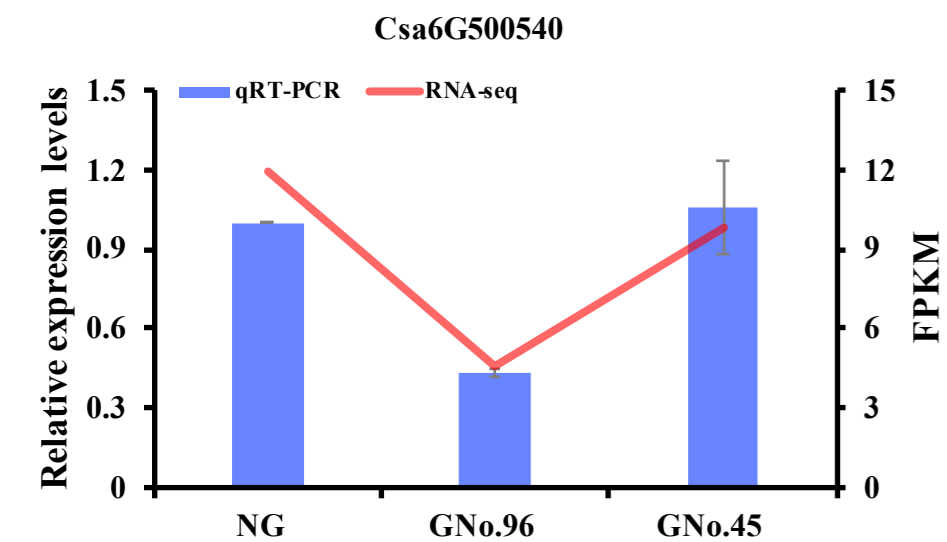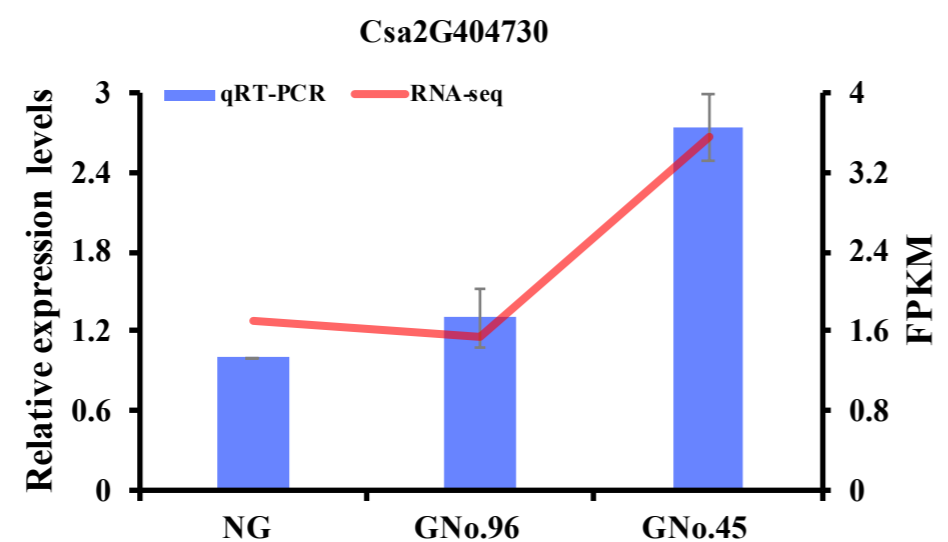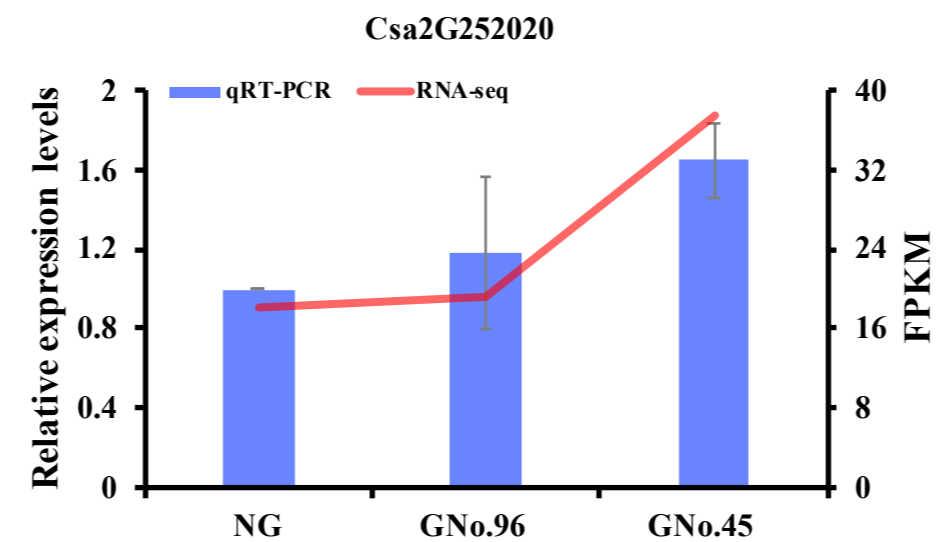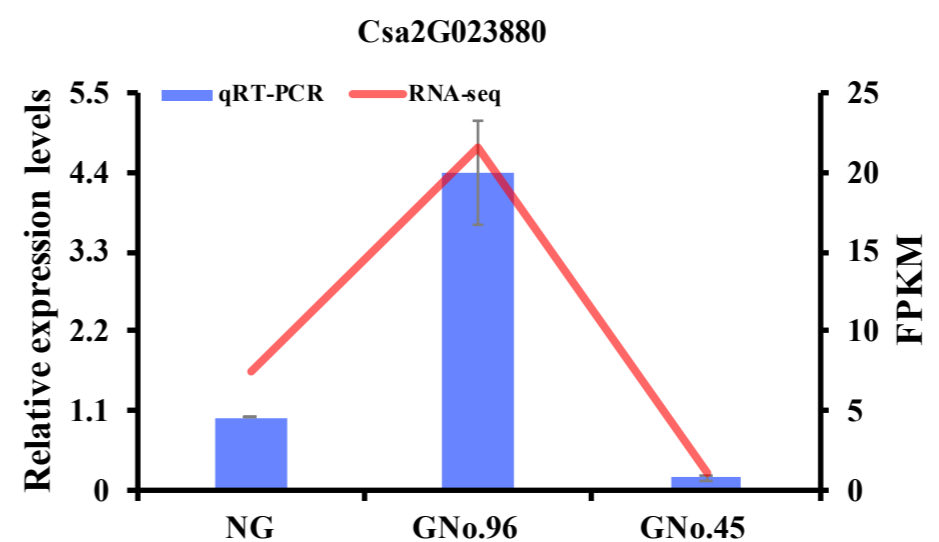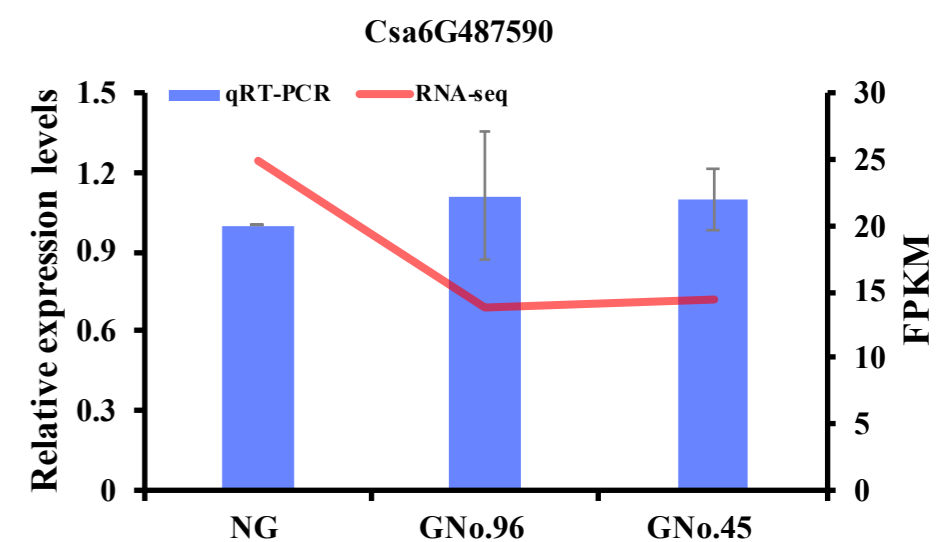

Supplement: Supplementary file 1 [file ijms-20-03592-s001.zip › supplement data/Figure S2.pdf]
